# A comparative study of Danhong injection and *Salvia miltiorrhiza* injection in the treatment of cerebral infarction: A systematic review and meta-analysis

**DOI:** 10.1097/MD.0000000000007079

**Published:** 2017-06-02

**Authors:** Kaihuan Wang, Dan Zhang, Jiarui Wu, Shi Liu, Xiaomeng Zhang, Bing Zhang

**Affiliations:** Department of Clinical Pharmacology of Traditional Chinese Medicine, School of Chinese Materia Medica, Beijing University of Chinese Medicine, Beijing, China.

**Keywords:** cerebral infarction, Danhong injection, meta-analysis, *Salvia miltiorrhiza* injection, systematic review

## Abstract

**Background:**

To evaluate systematically the clinical effectiveness and safety of Danhong injection (DI) and *Salvia miltiorrhiza* injection (SMI) in the treatment of cerebral infarction.

**Methods:**

A literature search was conducted for retrieving randomized controlled trials (RCTs) on cerebral infarction treated by Danhong injection and SMI in the Cochrane Library, PubMed, Embase, China Biology Medicine disc, China National Knowledge Infrastructure Database, China Science and Technology Journal Database, Wanfang Database up to January 22, 2017. Two reviewers extracted information and independently assessed the quality of included RCTs by the Cochrane Risk of Bias Assessment Tool; then data were analyzed with Review Manager 5.3 software.

**Results:**

Twelve RCTs involving 1044 patients were included. The result of DI group was about 27% superior to SMI group in the clinical total effective rate (relative risk 1.27, 95% confidence interval 1.19–1.35, *P* < .00001). In addition, DI could prefect neurologic impairment (standardized mean difference −1.22, 95% confidence interval −1.90 to −0.54, *P* = .0004), and adjust hemorheological parameters. Three RCTs occurred 4 cases of adverse drug reactions/adverse drug events, but there were no serious adverse drug reactions/adverse drug events.

**Conclusion:**

Comparing with SMI combined with western medicine, DI combined with conventional therapy is more effective in improving the clinical total effective rate and neurologic impairment, but more evidence-based medicine research needed to support our study further.

## 1 Introduction

Cerebral infarction, a common kind of cerebrovascular disease, is also referred to as ischemic stroke, which is resulted from cerebral blood circulation disorder, and hypoxia and ischemia. Cerebral infarction is inclined to attack in rest or sleep in most cases, and may reach peak hours within 2 days after outbreak; thus, cerebral infarction has the characteristics of high mortality and disability rate.^[1–3]^ In addition, cerebral infarction is one of the major diseases leading to death, whose incidence increases with age.^[4]^

In terms of Traditional Chinese Medicine (TCM) theory, cerebral infarction pertains to “apoplexy,” primarily due to blood stasis syndrome, and the therapeutic principle is promoting blood circulation to remove blood stasis of TCM.^[5]^ Recently, pharmacology experiments manifested that cerebral infarction involved certain pathologic changes, such as microcirculation disturbance, inflammatory response, vascular endothelial injury, and so forth. Among western medicine (WM) therapies, recent studies demonstrated that intravenous thrombolysis and endovascular thrombectomy have good effect on treating cerebral infarction; however, time window, correct diagnosis, anesthesia, treatment cost, and such problems need to be considered in the process of treatment, which may influence the desired effect. ^[6–8]^ Then, the therapeutic practice of activating blood circulation for removing blood stasis of TCM holds a significant position for the treatment of cerebral infarction.^[9]^ In addition, Chinese herbal injections (CHIs) own the features of remarkable curative efficiency, rapid action, and high bioavailability. Class for invigorating blood circulation CHIs is widely prescribed to patents with cerebral infarction in China.^[10,11]^ This study chose 2 representative CHIs, namely Danhong injection (DI) and *Salvia miltiorrhiza* injection (SMI), aiming to compare efficacy for cerebral infarction by gathering randomized controlled trails (RCTs) data.

Both DI and SMI are Danshen-based injections approved by the State Food and Drug Administration (SFDA) of China, which proved had effects on decreasing plasma viscosity, improving blood flow volume, and preventing platelet aggregation.^[12–14]^ As for ingredient, SMI is made of the extraction of Danshen (*Radix Salviae miltiorrhizae*), and DI is made of Danshen and Honghua (*Flos Carthami tinctorii*) extraction. Previous meta-analysis showed that DI made a significant influence on remedying cerebral infarction with less adverse drug reactions (ADRs); SMI is one of the most common injections for cerebral infarction as well.^[15]^ There is a lack of meta-analysis on evaluating DI and SMI directly, and to research their effectiveness further, we conducted a meta-analysis to access existing clinical evidence, to generalize application and provide clinical reference of DI and SMI.

## 2 Methods

### 2.1 Data sources and filtration strategy

A general search of published literature was conducted in the electronic databases from inception to January 22, 2017. Two reviewers (K.W. and D.Z.) independently searched RCTs and compared DI and SMI directly in the Cochrane Library, PubMed, Embase, China Biology Medicine disc, China National Knowledge Infrastructure Database, China Science and Technology Journal Database, and Wanfang Database without any restriction in languages. In Chinese databases, the keywords were: [“cerebral infarction” or “stroke” or “cerebral apoplexy” or “brain infarction” or “brain infarct” or “brainstem infarctions” or “cerebral thrombosis” or “cerebral embolism”] and [“Danhong injection” or “DanhongZhusheye” or “Beitong” or “ZhusheyongDanhong”] and [“*Salvia miltiorrhiza* injection” or “*Salvia miltiorrhiza* Zhusheye” or “Zhusheyong Danshen”]. In English databases, the method combining subject words and free words was applied into the retrieval. “Brain Infarction” was regarded as the Mesh term in the first retrieval, and the key words about injections were searched secondly in the results above. All the strategies were adapted into various forms with different databases. The strategy of PubMed are listed as follows:

(1) “Brain Infarction” [Mesh]
(2) “Cerebral Infarction” [Title/Abstract] OR “Stoke∗” [Title/Abstract] OR “Brain Embolism” [Title/Abstract] OR “Ischemic Stroke” [Title/Abstract] OR “Cerebrovascular Disorders” [Title/Abstract]
(3) (1) OR (2)
(4) “Danhong injection” [Title/Abstract]
(5) “*Salvia miltiorrhiza* injection” [Title/Abstract] OR “Danshen injection” [Title/Abstract]
(6) (3) AND (4) AND (5)

### 2.2 Inclusion and exclusion criteria

Randomized controlled trials conformed to inclusion as follows can be involved in this meta-analysis: clinical RCTs regarding cerebral infarction treated by Danhong injection and SMI whether use blinding or not. All patients were diagnosed as having cerebral infarction, and this was confirmed by head computed tomography (CT)/magnetic resonance imaging (MRI), in addition to accordance with the diagnostic standard “all kinds of cerebrovascular disease diagnosis” revised by Chinese medical association in 1995 at the fourth session of the national cerebrovascular conference.^[16]^ Included patients had no limitation of age, sex, race, and the severity of disease. In the implementation of RCTs, all patients were admitted to hospital for regular WM therapy, for instance, the way of anticoagulation, antiplatelet aggregation, lowering the intracranial hypertension, and perfecting brain metabolism. DI group injected DI and WM, SMI group injected SMI and the same WM combined SMI with the same WM. Beyond that, patients with other complications were given corresponding treatment. The criterion of therapeutical efficiency was congruent with “the norm of clinical neurologic deficit score” (CNDS) issued by Chinese Neuroscience Society in 1995.^[17]^ Clinical total effective rate and neurologic deficit scores were dominating outcomes, computed by National Institute of Health Stroke Scale (NIHSS) or CNDS: neurologic deficit scores which decreased by 91% to 100% belonged to the class of basic recovery, from 46% to 90% was classified into the part of remarkable progress, from 18% to 45% pertained to grade of progress, from 0% to 17% was counted as invalidation, and under 0 was deemed as deterioration. The clinical total effective rate was calculated by the formula: (number of basic recovery patients + number of remarkable progress patients + number of progress patients)/total number of patients × 100%. Secondary outcomes of this meta-analysis were hemorheology indexes (including plasma viscosity, content of fibrinogen, and so on), and content of total cholesterol, high-sensitivity C-reactive protein, and serum Hcy level, ADRs/adverse drug events (ADEs), and so forth.

Exclusion criteria were as follows: WM therapies contained rehabilitation and physical therapies; interventions involved other TCM treatment, for example, Chinese herbal medicine, acupuncture, and moxibustion; the literatures were without complete data or full text; as for the literatures with the same data, the one which had large sample size and relative complete information was retained; the self-controlled study and the RCTs with error randomized method.

### 2.3 Data extraction and quality assessment

While reading the literature titles and abstract independently, 2 researchers (K.W. and S.L.) used NoteExpress (Wuhan University Library, Wuhan, China) to manage literatures and filter out uncorrelated literatures, reviews, and pharmacology experiments. Then, the rest of the RCTs were read full text to identify whether it meet with the included criteria or not. As for included studies, researchers extracted the following necessary information: the basic information of study: first author, publication date; the features of patients: the number of DI and SMI group, sex proportion, age, intervention, dosages, and course of treatment; the measure data of outcomes of each RCT; the type of research designed and key factors of risk assessment. The Cochrane Risk of Bias Assessment Tool was adopted to assess quality of literatures, whose items contains sequence generation (selection bias), allocation concealment (selection bias), blinding of patients and personnel (performance bias), blinding of outcome assessment (detection bias), incomplete outcome data (attrition bias), selective outcome reporting (reporting bias), and other sources of bias.^[18]^ Each item was classified into 3 ranks: “low,” “high,” and “unclear.” “Low” risk referred to carrying out an appropriate random method and blinding. “High” risk referred to an incorrect method and no blinding. “Unclear” referred to no description on details about risk assessment. If there was any divergence between the 2 researchers, the third person (J.W.) should resolve disagreement.

Ethical approval was unnecessary for this meta-analysis, because our meta-analysis was the procedure that just gathered the experimental data in each RCT without any leak of patient information.

### 2.4 Statistical analysis

All the meta-analyses data utilized Review Manager 5.3 (Cochrane Collaboration, Oxford, UK) to synthesize and analyze.^[19]^ For outcomes, this meta-analysis chose relative risk (RR) to evaluate dichotomous outcomes, while using standardized mean difference (SMD) and mean difference (MD) to assess continuous variables; each outcome numerical value was presented with 95% confidence intervals (95% CIs) as well. Besides, forest plots also demonstrated the results between the 2 groups. Heterogeneity between RCTs was analyzed by chi-square test and estimated by *I*^*2*^. Meta-analyses were calculated by random-effects model.^[20]^ In addition, a funnel chart was drawn to analyze the potential publication bias. Egger test and Begg test were also utilized to asses publication bias, and *P* < .05 was deemed as statistically significant.^[21]^ Furthermore, to test the stability of results, the sensitivity analysis was conducted in clinical total effective rate by STATA 12.0 (StataCorp LP, College Station, TX).

## 3 Results

### 3.1 Study characteristics

**Figure 1:**
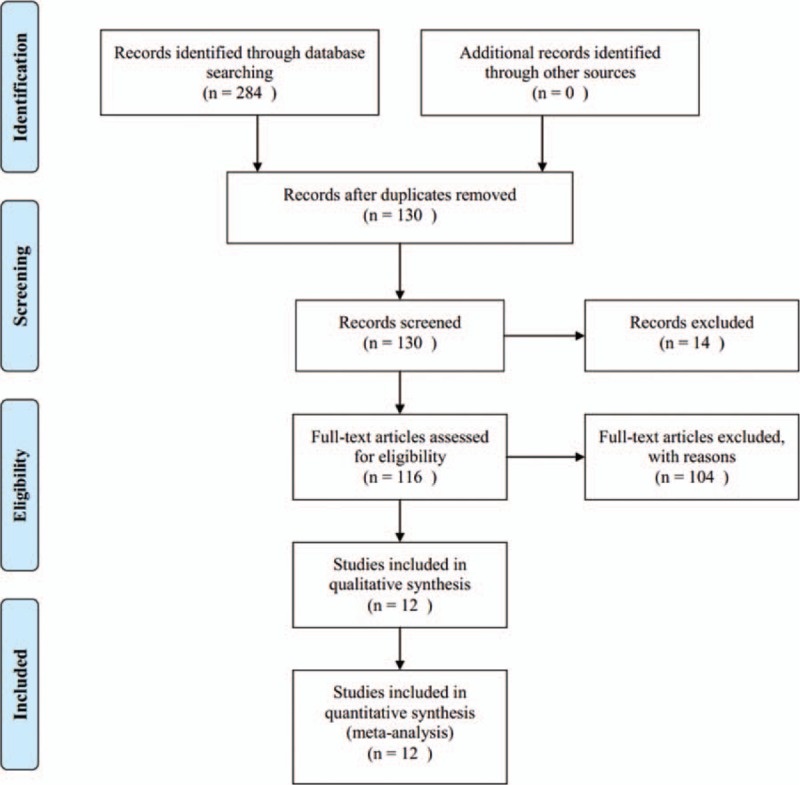
Flow chart of literature search.

The literature search flow was depicted in Fig. 1. We retrieved 284 literatures during the initial selection. After reading titles and abstracts, duplications, irrelevant articles, and reviews were excluded. Then, we obtained 116 RCTs on comparing DI and SMI for treating cerebral infarction. After reading the full text, a total of 100 RCTs were excluded: in 8 RCTs, patients were not diagnosed as having cerebral infarction, 81 did not comply with the intervention of inclusion criteria, 13 did not refer to the diagnostic standard or therapeutical criterion, 1 was not performed in correct randomized method, and in 1 RCT, clinical data were identical with the other one. Finally, 12 Chinese RCTs were identified in meta-analysis, which comprised of 526 patients in the experimental group and 518 patients in the control group. All patients were diagnosed as having cerebral infarction by the diagnostic standard, among which male patients accounted for 57.1% (596/1044). In the 12 RCTs, the maximum sample size was 182 cases, whereas the minimum sample size was 40 cases. As for intervention, the experimental group was DI and WM and the control group was SMI and WM; WM included aspirin, defibrase, venoruton, and so on. Patients received treatment once a day, and the period of treatment was almost 14 days. The studies and patient characteristics are presented in Table 1.

**Table 1:**
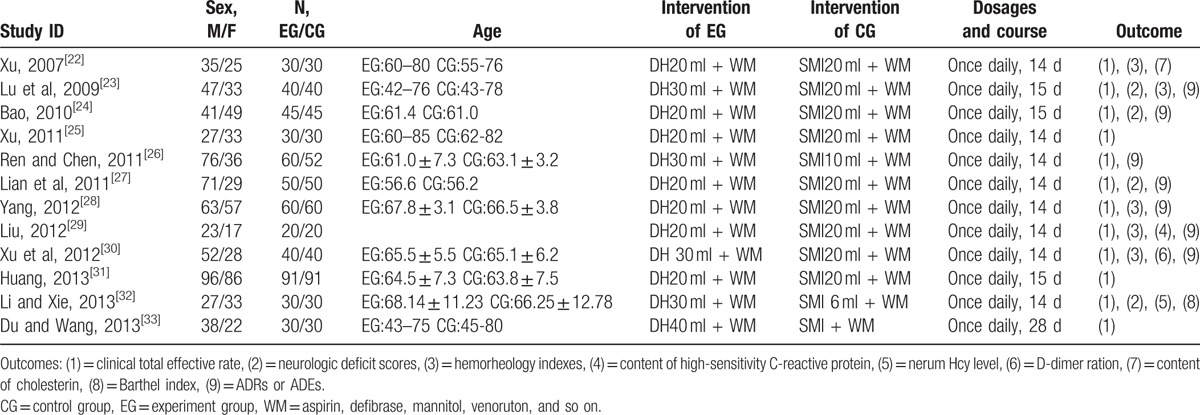
Study characteristics.

**Figure 2:**
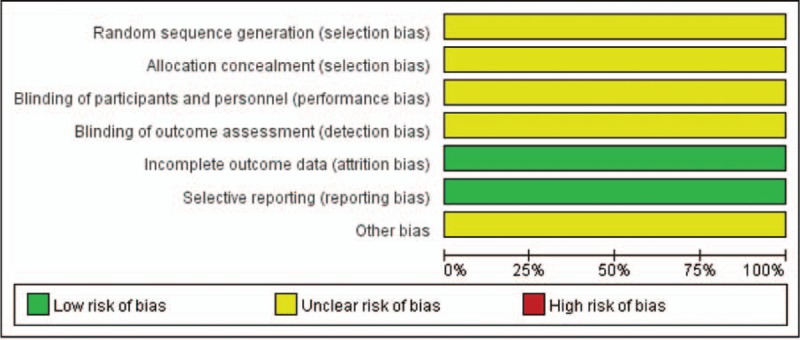
Risk of bias summary.

This meta-analysis used the Cochrane Risk of Bias Assessment Tool to perform quality assessment. The 12 RCTs included merely mentioned dividing patients into 2 groups randomly, and did not illustrate how to implement randomized method and conceal allocation; therefore, selection bias was evaluated as “unclear risk.” Because none of the included studies was conducted in blinding, performance bias and detection bias were evaluated as “unclear risk.” Besides, case deficiency or selective reporting was not present; hence, attrition bias and reporting bias were assessed as “low risk.” Regarding other biases, 12 RCTs did not offer any details contributing to high risk, so other biases were evaluated as “unclear risk.” Graphical description is shown in Fig. 2.

### 3.2 Outcomes

#### 3.2.1 Clinical total effective rate

**Figure 3:**
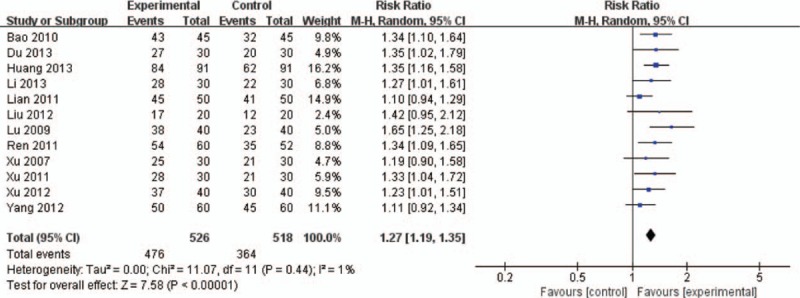
Meta-analysis in clinical total effective rate between DI + WM and SMI + WM. DI = Danhong injection, SMI = *Salvia miltiorrhiza* injection, WM = western medicine.

Pooling of the data from the 12 RCTs including for clinical total effective rate and the random-effects model was used.^[22–33]^ Meta-analysis results indicated a statistically significant difference between DI group and SMI group. With the same combination with WM treatment, DI can be about 27% superior to SMI in improving clinical total effective rate (RR 1.27, 95% CI 1.19–1.35, *P* < .00001; Fig. 3).

#### 3.2.2 Sensitivity analysis and publication bias

**Figure 4:**
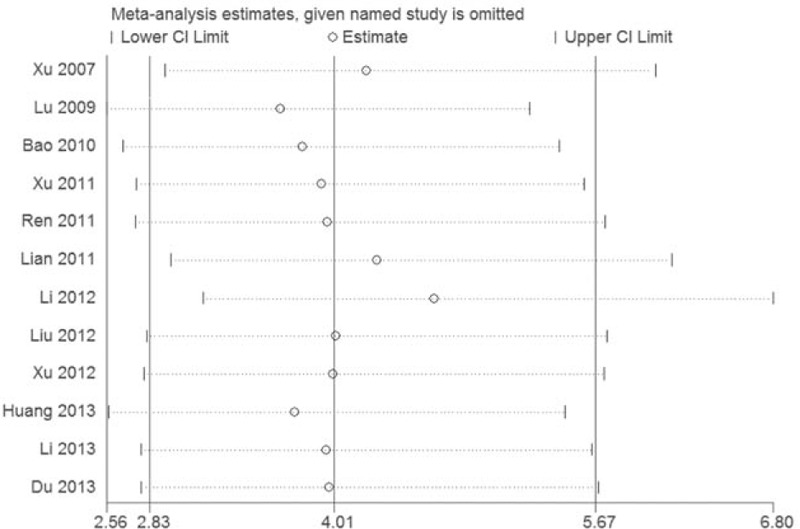
Sensitvity analysis in clinical total effective rate.

For clinical total effective rate, we carried out a sensitivity analysis to verify the independence of result, which was done by excluding the RCT seriatim at a time to re-synthesize the data. As can be seen from Fig. 4, clinical total effective rate had no qualitative change, and this result had a good stability.

**Figure 5:**
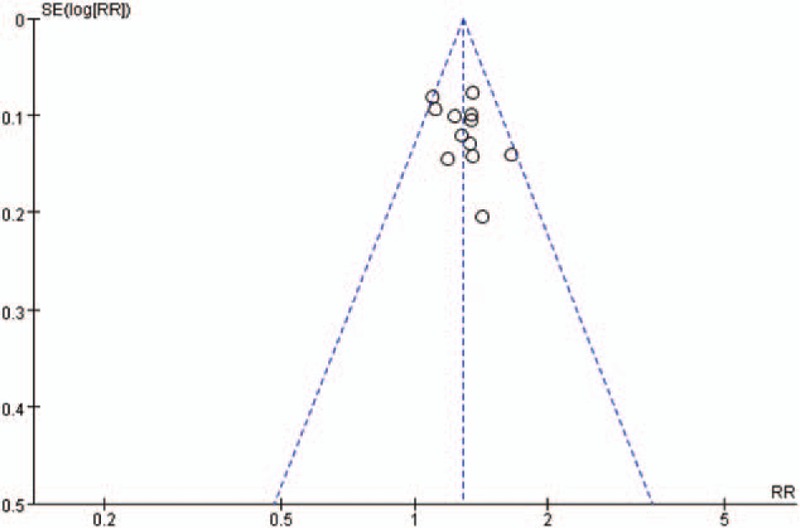
Funnel plot of publication bias.

Figure 5 displayed a funnel plot on publication bias for clinical total effective rate, which was depicted by RR values and the standard error of RR values. The funnel plot presented a general symmetry, and the RCTs included concentrated upon the upper part of it. Moreover, the results of Egger test (*t* = 0.47, *P* = .174 > .05) and Begg test (*z* = 0.62, *P* = .537 > .05) indicated no evidence of significant publication bias.

#### 3.2.3 Neurologic deficit situation

**Figure 6:**
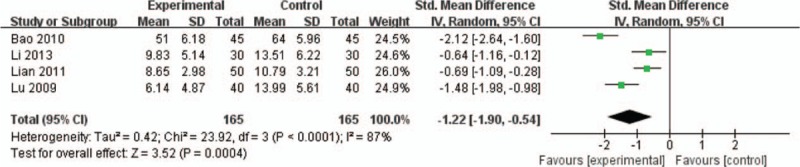
Meta-analysis in neurologic deficit situation between DI + WM and SMI + WM. DI = Danhong injection, SMI = *Salvia miltiorrhiza* injection, WM = western medicine.

In all, 4 RCTs referred to this outcome.^[23,24,27,32]^ Meta-analysis result demonstrated a statistically significant difference between DI group and SMI group; thus, in terms of improving neurologic deficit situation, a combination of DI and WM was prior to the conjunctive use of SMI and WM (SMD −1.22, 95% CI −1.90 to −0.54, *P* = .0004; Fig. 6).

#### 3.2.4 Hemorheology indexes

In all, 5 RCTs mentioned hemorheology indexes, including plasma viscosity, content of fibrinogen, whole blood high-shear viscosity, and whole blood low-shear viscosity. More details are presented in Table 2.

(1) Plasma viscosity: In all, 5 RCTs referred to plasma viscosity.^[22,23,28,29]^ The result of meta-analysis signified that there was no statistically significant difference between the 2 groups, so both DI and SMI could have a good impact on decreasing plasma viscosity.
(2) Content of fibrinogen: There were 3 RCTs involved in the content of fibrinogen.^[23,29,30]^ Meta-analysis result showed that there was a statistically significant difference between the 2 groups with respect to fibrinogen's content, and a combination of DI and WM was more effective than control group.
(3) Whole blood high-shear viscosity: There were 2 RCTs which mentioned the whole blood high-shear viscosity.^[22,29]^ Meta-analysis result indicated that there was a statistically significant difference between the 2 groups, and the conjunctive use of DI and WM could lower the whole blood high-shear viscosity significantly than control group.
(4) Whole blood low-shear viscosity: A total of 2 RCTs referred to the whole blood low-shear viscosity.^[22,29]^ Meta-analysis result manifested that there was a statistically significant difference between the 2 groups, and the combination of DI and WM could achieve a better effect on reducing the whole blood low-shear viscosity.

**Table 2:**
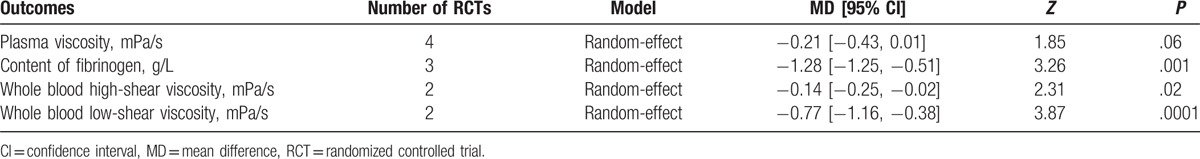
Meta-analysis of hemorheology indexes.

### 3.3 Other outcomes

There were 4 RCTs which reported the serum Hcy level, content of high-sensitivity C-reactive protein, and other secondary outcomes, respectively^[22,29,30,32]^; thus, this study made a qualitative description for those indexes. More details are presented in Table 3.

**Table 3:**
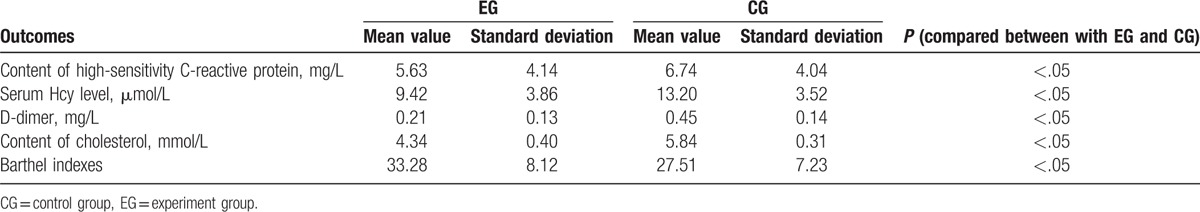
Meta-analysis of other outcomes.

### 3.4 Safety

A total of 4 RCTs mentioned there were no obvious ADRs/ADEs during the implementation of trials.^[24,26,29,30]^ One RCT occurred 1 case of skin itch and 1 case of erubescence in the experimental group, patients could tolerate and alleviate by themselves without treatment.^[27]^ There were 2 RCTs which reported ADRs/ADEs: 1 case of rash fever and 1 case of low-grade fever, both of which recovered by themselves.^[23,28]^ The rest of the 5 RCTs did not report about ADRs/ADEs.^[22,25,31,32,33]^

## 4 Discussion

According to the results of this meta-analysis, DI can make a more noticeable impact than SMI for cerebral infarction patients, which was embodied in the following aspects: first, a combination use of DI and WM has a notable performance on improving clinical total effective rate, perfecting neurologic deficiency, lessening the content of fibrinogen, and decreasing whole blood high-shear viscosity and whole blood low-shear viscosity. Moreover, compared with an integration of SMI and WM, DI combined with WM can lower the serum Hcy level, reduce the content of high-sensitivity C-reactive protein, cholesterol, and D-dimer, and increase Barthel indexes.

The results of this meta-analysis were consistent with the pharmacologic action of DI. DI is manufactured by the extractive of Danshen (*Radix Salviae miltiorrhizae*) and Honghua (*Flos Carthami tinctorii*) under the guidelines of TCM, whose effective constituents are tanshinone, salvianolic acid, safflower yellow, safflower phenolic glycosides, catechol, and so on. Danshen has the characteristics of bitter taste and slight cold nature, also deemed to monarch drug in this prescription due to its function that scatters stasis and unclogs arteries. Honghua owns the traits of pungent taste and slight cold nature, and its functions were dispersing blood stasis and dredging collateral, which plays an auxiliary part in this prescription. These 2 herbs act together to make a contribution that eliminate pathogenic factors and defend healthy “*qi*,” especially suitable for treating cerebral infarction and other blood stasis stagnation syndromes.^[34]^ The results of pharmacological experiments manifested that DI owns a capacity not only on antiplatelet aggregation, anticoagulation, and decreasing blood viscosity, but also upon regulating blood lipid and boosting the activity of fibrinogen dissolved, which make a dedication that perfect cerebral microcirculation, nourish brain tissue, and prevent thrombus enlargement for cerebral infarction patients. Apart from that, the other crucial reason that DI is widely used in treating CI is that DI has a pharmacologic action on protecting neurons and inhibiting the inflammatory response, which is vital for cerebral infarction patients’ prognosis.^[35,36]^ In contrast with SMI, the effect of resisting cerebral ischemic injury and anti-inflammatory action of DI is enhanced.^[37]^

As for ADRs/ADEs, because most RCTs did not report ADRs/ADEs, so our study could not draw a definite conclusion about security of DI. Though the occurrence proportion was low in this meta-analysis, the use of DI should be paid more attention to. Researchers suggested that the ADRs/ADEs of DI almost happened within 30 minutes after injecting, and affected multiple organs, mainly skin and its accessories. The reason that DI may increase ADRs/ADEs was associated with sensitinogen present in it, such as pollen protein, tanshinone, and tannin, which can bond with plasma proteins and then lead to anaphylactic reaction.^[38]^ Therefore, medical staff should use DI according to instructions, and more specifically concentrate on ADR/ADE supervision. Besides, individual differences between patients should be focused on in clinical application, particularly the patients who have a history of allergy.^[39]^

At present, there is a lack of systemic reviews about comparing DI and SMI in the treatment of cerebral infarction directly in databases. Hence, this meta-analysis had the following 3 advantages: firstly, both DI and SMI were common CHIs in treating cerebral infarction; this meta-analysis made an evaluation in efficiency and safety about its progress and evidence supplement. Secondly, we combined subject words with free words in retrieval and then made a comprehensive search in the Cochrane Library, PubMed, Embase, China Biology Medicine disc, China National Knowledge Infrastructure Database, China Science and Technology Journal Database, and Wanfang Database. Thirdly, in terms of inclusion and exclusion principles, this meta-analysis formulated a relative strict inclusion that the diagnostic standard, the therapeutical criterion, and interventions were congruent to ensure the identical base line, which was vital to decrease the impact result from clinical heterogeneity. Meanwhile, this meta-analysis excluded the RCTs in which randomized sequences were generated as per patients’ hospitalization, aiming to guarantee the quality of included studies.

### 4.1 Limitations

This meta-analysis also had limitations. First, the quality of overall RCTs was general. All included studies mentioned “random” in RCTs and did not make a detailed description about how to generate random sequence, conceal allocation, or whether carried out blinding, which may bring about certain bias for assessment and influence the grade of evidence. Secondly, though Egger test and Begg test showed that there was no publication bias in this study, the included RCTs concentrated upon the upper part of funnel plot. It revealed that our meta-analysis may lack RCTs whose sample size was small and quality was high. Apart from that, the treatment course of included RCTs was short and clinicians did not conduct follow-up visit; thus, we could not judge whether there was any significant difference between the 2 interventions about recurrence rate or mortality of patients. As for outcomes, secondary outcomes in this meta-analysis were significant as well, but there were several RCTs which referred to them, for instance, Barthel indexes can reflect the quality of patients’ survival directly, D-dimer also can be regarded as an indicator of curative effect and prognosis situation. Despite the above limitations, our study provided a complete evaluation for the effectiveness and safety of DI and SMI on treating cerebral infarction.

## 5 Conclusions

To sum up, our study made a comparison on effectiveness and safety between DI and SMI. The results manifested that DI was more effective than SMI on improving clinical total effective rate, perfecting neurologic deficit situation, and other aspects. However, we proposed several suggestions based on foregoing limitation: firstly, RCTs should be registered in advance and implemented according to Consort standard so as to ensure the transparency of trial process. Personnel ought to conceal random sequence allocation and carry out blinding as possible.^[40]^ Secondly, clinicians had better chose mortality, recurrence rate, patients’ survival quality evaluation and other long-term outcomes correlated closely with patients as indicators. Thirdly, it is the responsibility of the medical staff to use DI as per the instruction guidelines and monitor occurrence of ADRs/ADEs. In general, we draw a conclusion that DI had a positive effect on treating cerebral infarction, but more multicenter and high-quality RCTs should be implemented in the future to support evidence.
